# HIV-1 diversity in Cameroon: new insights on the evolution of the virus

**DOI:** 10.1186/1742-4690-9-S2-P147

**Published:** 2012-09-13

**Authors:** T Aime Marcel Simon, Z Lycias, M Eitel, W Carolyn, BA Wendy, MP Darren

**Affiliations:** 1 Institute of Infectious Disease and Molecula, University of Cape Town, Cape Town, South Africa; 2 IIDMM, University of Cape Town, Cape Town, South Africa; 3 Institute of Medical Research and Study of Medicinal Plants, Yaounde, Cameroon

## Background

Given the central role of HIV-1 diversity in the HIV pandemic and its impact on vaccine development, it is imperative that global molecular epidemiology surveillance continues. Constantly improving phylogenetics-based analytical techniques and rapidly expanding HIV sequence datasets promise to yield important insights into the origin, evolution and spread of HIV-1.

## Methods

In an effort to study the phylogeography and phylodynamics of the HIV-1 M epidemic in the early 20th century, we analysed 50 plasma samples from HIV-infected blood donors from Cameroon. Full length gag sequences were generated and aligned using MUSCLE along with a representative selection of HIV sequences from the rest of the world and all published gag sequences from Cameroon and other West African countries. A maximum likelihood phylogenetic tree was constructed from these sequences following removal of recombinant sequence fragments by a blinded fully exploratory screen for recombination using RDP3.

## Results

All the Cameroonian sequences were derived from HIV-1 M viruses. The phylogenetic tree indicated that at least one of the newly-sequenced CRF02_AG viruses is an outlier of the CRF02_AG clade and may help resolve the controversy surrounding the origins of this clade. Furthermore, isolates from Cameroon were spread throughout the phylogenetic tree clustering with different subtypes and circulating recombinant forms, a finding consistent with West Africa being the geographic origin of the global HIV epidemic. Importunately, our blind recombination screen suggested that many divergent Cameroonian viruses previously identified as being unique recombinant forms, may be divergent, relatively non-recombinant, but under-sampled subtype-level lineages.

## Conclusion

Lineages diverging early after the origin of HIV-1 M are likely still circulating in Cameroon and could be suitable for retracing the movement and evolutionary dynamics of HIV-1 during the earliest stages of the pandemic. These lineages will be useful for reconstructing ancestral HIV-1M sequences for use as vaccine immunogens.

